# UNDERSTANDING THE ROLE OF SOCIAL SUPPORTS AND SOCIAL NETWORK FOR DEMENTIA CAREGIVERS’ MENTAL HEALTH

**DOI:** 10.1093/geroni/igz038.492

**Published:** 2019-11-08

**Authors:** Carmen Morano, daejun park, Andrea Savage

**Affiliations:** 1 School of Social Welfare, University of Albany, State University of New York, Albany, New York, United States; 2 University at Albany, School of Social Welfare, Albany, New York, United States; 3 Silberman School of Social Work, New York, United States

## Abstract

This paper explores the associations of depressive symptoms with social supports and social networks among dementia caregivers. It has been well documented that dementia caregivers are at greater risk of experiencing negative mental health and poorer physical health than non-caregivers. This paper describes a collaborative process between two universities and a community-based provider in designing a Social Network Analyses to examine the network structures used by dementia caregivers participating in a community-based support program. The relationship between the caregiver support networks and depressive symptoms, were analyzed using multivariate regression models. Given the small sample size and missing data multiple imputation was applied to the data. The findings suggest the effects of a variety of supports in the caregiver network on mental health and depressive symptoms. Among the findings it was found that the presence of financial support (B= - 0.58; p = .01) and frequency of contacts (B = -0.58; p = .01) support resulted in a decrease in depressive symptoms and better mental health than for caregivers without similar supports in their networks. This paper will conclude with a discussion of potential uses of social network analysis to better understand how the structure of caregivers’ network can address the concrete physical, emotional and financial needs of dementia caregivers.

